# The relationship between creamatocrit and cumulative percentage of total milk volume: a cross-sectional study in mothers of very preterm infants in Bangkok, Thailand

**DOI:** 10.1186/s13006-023-00599-5

**Published:** 2023-11-23

**Authors:** Walaiporn Bowornkitiwong, Chulaluk Komoltri, Sopapan Ngerncham

**Affiliations:** 1 Division of Neonatology, Department of Pediatrics, Faculty of Medicine Siriraj Hospital, Mahidol University, Bangkok, 10700 Thailand; 2 Division of Clinical Epidemiology, Department of Health Research and Development, Faculty of Medicine Siriraj Hospital, Mahidol University, Bangkok, Thailand

**Keywords:** Creamatocrit, Foremilk, Hindmilk, Preterm infants, Thai

## Abstract

**Background:**

Human hindmilk contains higher concentrations of fat than foremilk and is more desirable for growth in preterm infants who can tolerate limited volumes of breastmilk. There is currently no clear demarcation between foremilk and hindmilk. This study characterized the change in breastmilk’s fat content from the start to end of milk flow and defined this demarcation.

**Methods:**

Mothers of infants born at ≤ 32 weeks gestational age and ≥ 14 days after childbirth in a University hospital in Bangkok, Thailand between July, 2011, and April, 2012 were included in this cross-sectional study. Breastmilk samples were sequentially collected from the start to end of milk flow in 5-mL aliquots using breast pumps. The fat content of each aliquot from each breast was determined through creamatocrit. The average creamatocrit of foremilk and hindmilk were compared in predefined foremilk to hindmilk ratios of 20:80, 25:75, 33:67, and 50:50. Creamatocrit of the first and last aliquots were compared for mothers who expressed low- (≤ 25-mL per breast) and high-volumes (> 25-mL per breast) of breastmilk.

**Results:**

Of the 25 mothers enrolled, one was excluded due to unsuccessful creamatocrit measurement. The last aliquot of breastmilk had a significantly higher creamatocrit than the first from the same breast (median [interquartile range] of 12.7% [8.9%, 15.3%] vs. 5.6% [4.3%, 7.7%]; test statistic 1128, *p* < 0.001). Mean creamatocrit in hindmilk portions (9.23%, 9.35%, 9.81%, and 10.62%, respectively) was significantly higher than foremilk portions (6.28%, 6.33%, 6.72%, and 7.17%, respectively) at all predefined ratios. Creamatocrit increased by 1% for every 10% incremental increase in expressed breastmilk volume until the breast was emptied. Low-volume mothers had a significantly higher creamatocrit in the first aliquot compared with high-volume mothers (U = 437, *p* = 0.002). No significant difference in breastmilk volume was observed between mothers with and without breastfeeding experience.

**Conclusions:**

Fat content in breastmilk increased on an incremental basis. More fluid definitions of foremilk and hindmilk should be adopted. Mothers should prepare their breastmilk into aliquots based on the required feeding volume of their infant. Hindmilk aliquots can be prioritized over foremilk aliquots to ensure infants obtain optimal caloric intake.

**Supplementary Information:**

The online version contains supplementary material available at 10.1186/s13006-023-00599-5.

## Background

Human breastmilk is regarded as the best source of nutrition for newborns. Enteral feeding with breastmilk is associated with reduced necrotizing enterocolitis, late-onset sepsis, retinopathy of prematurity, bronchopulmonary dysplasia, and neurodevelopmental impairment in preterm very low birth weight (VLBW) infants [1–3]. However, breastmilk alone does not meet the nutritional requirements of VLBW infants and should be fortified to optimize VLBW infants’ growth [2, 4].

Fat constitutes approximately 3–5% of human breastmilk and serves as the primary source of energy for infants [5]. This fat content can vary significantly for different mothers, as well as the same individual depending on the time of day and breastfeeding session [5–7]. During breastfeeding or breastmilk expression, the initial portion of the milk stream (foremilk) contains higher levels of lactose but lower fat content compared with the latter portion of the stream (hindmilk) [5, 8, 9]. This disparity in composition between foremilk and hindmilk significantly impacts the physiology of infants’ digestion. Foremilk mainly hydrates infants and acts as a mild laxative, while the higher fat content in hindmilk provides high caloric value to promote growth as well as delay the transit time of breastmilk in infants’ gastrointestinal tracts [5, 10]. Infants only receive the full range of benefits that human breastmilk has to offer when they can breastfeed until the breast is emptied, and a maximum amount of hindmilk is ingested.

When VLBW infants are critically ill and hospitalized, the amount of breastmilk that infants can tolerate is limited. As foremilk is expressed first, this inadvertently results in infants consuming little hindmilk – leading to inadequate weight gain. Several neonatal intensive care units (NICUs) reported that hindmilk feeding improved growth in premature infants [11–14]. Previous literature explicated foremilk and hindmilk inconsistently, as summarized in Table 1 [11, 15–20]. Defining the demarcation between foremilk and hindmilk in a milk stream can appear arbitrary. There are currently no standardized guidelines that can be reliably used to calculate the optimal volume of foremilk to be expressed before a desired portion of hindmilk can be obtained. Despite the general acceptance that hindmilk contains a higher fat content, previous literature has not ascertained when foremilk becomes hindmilk. This ambiguity is particularly problematic in clinical practice when infants have a limited breastmilk intake volume and should primarily receive the portion that will help them thrive.

**Table 1:** Previous definitions of foremilk and hindmilk

Reference	Foremilk definition	Hindmilk definition	Note(s)	Gestational age (wk)
Valentine et al., 1994 [11]	Breastmilk collected 2–3 min after milk flow.	Remaining breastmilk collected through complete breast emptying.	Foremilk fraction comprised 40% of the total milk volume.	24–35
Uyan et al., 2005 [15]	Breastmilk collected 2–3 min milk after flow.	Remaining breastmilk collected during complete breast emptying.	Neither foremilk nor hindmilk is superior to the placebo in relieving pain (during heel prick).	Full-term
Bishara et al., 2008 [16]	Breastmilk collected 3 min after milk flow.	Remaining breastmilk collected until the breast is emptied.	Retinol, tocopherol, fat, energy, and nitrogen concentrations were significantly higher in hindmilk than foremilk.	< 28
Bishara et al., 2009 [17]	Breastmilk collected 3 min after milk flow.	Remaining breastmilk collected until milk flow ceases.	Foremilk volume comprised approximately 40% of the total milk volume.	< 28
Murase et al., 2009 [18]	Breastmilk collected from 3 nipple openings by hand expression before breastfeeding.	Breastmilk collected from three nipple openings by hand expression after breastfeeding.	The fat and protein content of foremilk and hindmilk differed across milk ducts despite being expressed from the same breast.	Full-term
van Sadelhoff et al., 2018 [19]	The first 30-mL of breastmilk after milk flow.	The remaining volume of breastmilk.	Hindmilk, compared with foremilk, had a higher total protein content but lower total free amino acid content.	Full-term
Takumi et al., 2022 [20]	Breastmilk collected before the breastfeeding session.	Breastmilk collected after the same breastfeeding session.	Hindmilk had a higher fat concentration than foremilk.	Full-term

Abbreviations: wk, week

Creamatocrit measurements are one means of assessing fat content in breastmilk, which is determined by centrifuging breastmilk in capillary tubes – akin to hematocrit measurements. The length of the fat and total milk columns can be measured using Vernier Calipers or a hematocrit reader, and the proportion of fat to total volume (creamatocrit) expressed in percentage [13, 18, 21, 22]. The use of centrifugal force to separate cream from milk may result in some fat remaining in the supernatant – hence, the slightly lower creamatocrit level compared with the gold-standard gravimetric method (which also indicates fat proportion) [21, 23, 24]. Regardless, creamatocrit remains a simple and reliable measurement that can be performed by nurses and non-medical personnel (mothers) alike [13, 21–23]. This proves extremely useful and practical in ascertaining the demarcation in breastmilk’s fat content from foremilk to hindmilk.

The primary objective of the study was to characterize the progressive changes in breastmilk’s fat content from the start to end of milk flow through creamatocrit. The secondary objective was to define a demarcation between foremilk and hindmilk.

## Methods

### Study design, setting, and participants

This cross-sectional study was conducted at the Division of Neonatology, Department of Pediatrics, Faculty of Medicine Siriraj Hospital, Mahidol University, Bangkok, Thailand between July 1, 2011 and April 30, 2012. Siriraj Hospital is the largest tertiary care center in Thailand, with approximately 8,000 childbirths per year. Mothers of infants who were born at ≤ 32 weeks gestational age and ≥ 14 days after childbirth were included in the study. This was to ensure that expressed breastmilk consisted of mature milk. Mothers who used domperidone as a galactogogue or with an expressed breastmilk volume < 5-mL were excluded. Eligibility assessments were performed by W.B. and a practical nurse responsible for overseeing the neonatal ward’s lactation room. Mothers who stayed overnight in the neonatal ward were invited and informed of the study by W.B. All provided written informed consent prior to undergoing any study procedures.

### Milk collection and creamatocrit measurements

Breastmilk from the start to end of milk flow from each breast was collected during the same breast pumping session. Breastmilk samples were collected in the lactation room of the neonatal ward where infants were admitted. Mothers were taught how to practice proper hygiene; this entailed thorough handwashing techniques with soap and water as well as how to clean their breasts with sterile water. All mothers used a Medela® electric breast pump (Medela AG, Switzerland; CE 0123) to collect their breastmilk in 5-mL aliquots into sequentially-labelled, sterile 5-mL bottles from the start to end of milk flow. Samples were separately collected from left and right breasts. Bottles collected towards the end of milk flow that had a volume < 5-mL were discarded. Breastmilk samples were kept at room temperature and creamatocrit measurements performed within 30 min of collection.

**Fig. 1:**
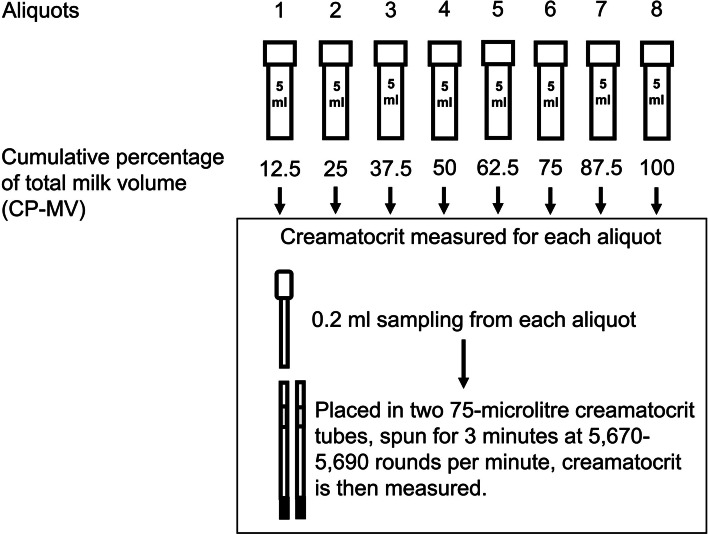
Breastmilk sample collection process. Illustrated above is a demonstration of how a 40-mL (8 tubes) volume of breastmilk from one breast would be processed after collection in 5-mL aliquot. The cumulative percentage of total milk volume (CP-MV) was calculated as an example

The proportion (in percentage) of breastmilk in each bottle was calculated based on the total expressed volume from each breast. The cumulative percentage of total milk volume (CP-MV) was calculated as shown in Fig. 1.

Creamatocrit measurements were performed by W.B. using Creamatocrit Plus® (Separation Technology, Inc. Florida, USA; ETL 9,700,686). Collection bottles were shaken to ensure thorough mixing before 0.2-mL of breastmilk was transferred from each bottle to two 75-µL creamatocrit tubes. These tubes were sealed with sealant before centrifugation in Creamatocrit Plus® for 3 min at 5,670-5,930 rounds per minute. Creamatocrit values were read thereafter using the machine-equipped reader tray and CP-MV calculated for each aliquot of breastmilk (as elaborated previously in Fig. 1)

## Statistical analyses

Statistical analyses were performed using SAS® Studio 9.2 (SAS® Institute Inc.) and IBM® SPSS® Statistics 28 (SPSS® Inc., Chicago, IL, USA). Descriptive statistics were used to present mothers’ demographic data. Creamatocrit values between the first and last sample aliquots from the same breast were compared using either paired t-tests or Wilcoxon signed-rank tests, depending on the data distribution.

Four random-coefficient mixed models (linear with random intercept, linear with random intercept and slope, quadratic with random intercept, and quadratic with random intercept and slope) were fit to examine the relationship between CP-MV of a breast (represented by X) and its corresponding creamatocrit (represented by Y). The model with the lowest Akaike information criteria (AIC) and Bayesian information criteria (BIC) was selected as the final model [25]. Regression diagnostics using residuals from the final model were tested; assumptions were not violated [25]. A minimum of three data points were required to test non-linear relationship. Mothers who had less than three aliquots of breastmilk per breast on either side were excluded from the analysis. Data from the breast with greater breastmilk volume was used in the analysis for each mother. Data from the right breast was used when both breasts expressed equal volume.

**Fig. 2:**
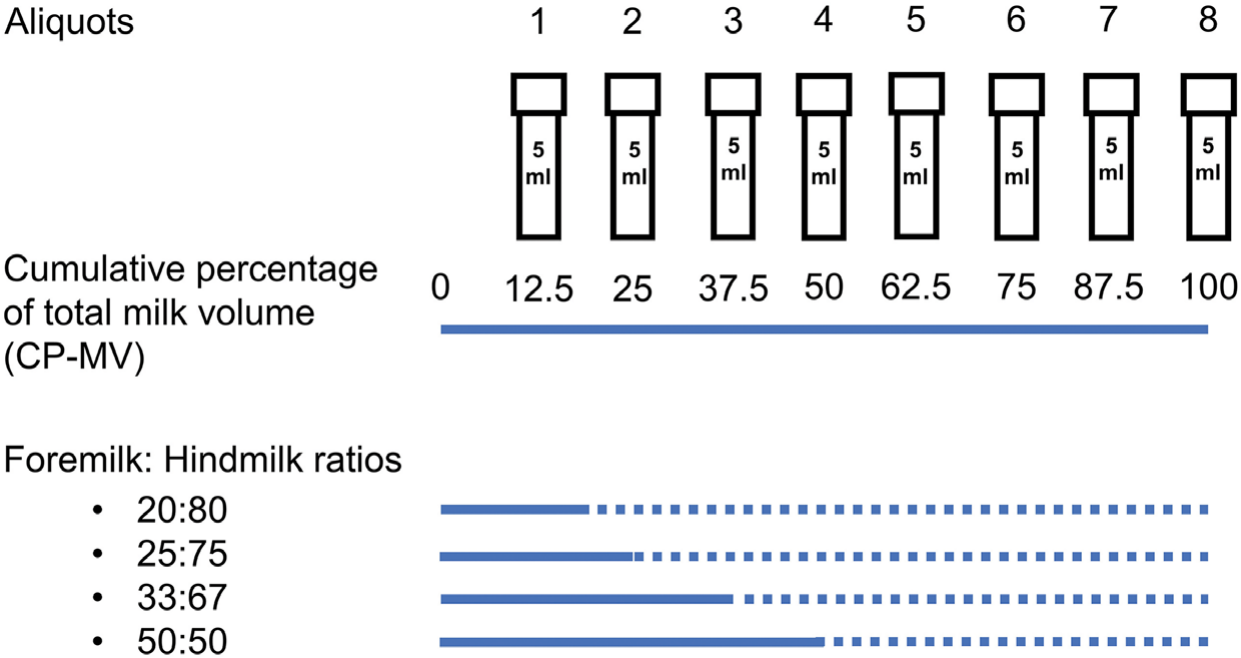
Foremilk to hindmilk ratios. Illustrated above is a continuation of how the previous 40-mL volume of human breastmilk from one breast would be used to calculate the cumulative percentage of total milk volume (CP-MV) as predefined ratios of 20:80, 25:75, 33:67, and 50:50 of foremilk:hindmilk

The researchers hypothesized that hindmilk started at the point where the fat content rose significantly. To define this point of demarcation in the breastmilk stream, the researchers arbitrarily determined the proportion of foremilk as 20%, 25%, 33% and 50% of the milk stream. This constituted 20:80, 25:75, 33:67, and 50:50 ratios of foremilk:hindmilk, respectively (as shown in Fig. 2). The researchers then used paired t-tests to compare the average creamatocrit of the tubes designated as foremilk and hindmilk to determine the most appropriate ratio (proportion with a significant rise in creamatocrit levels).

## Results

**Fig. 3:**
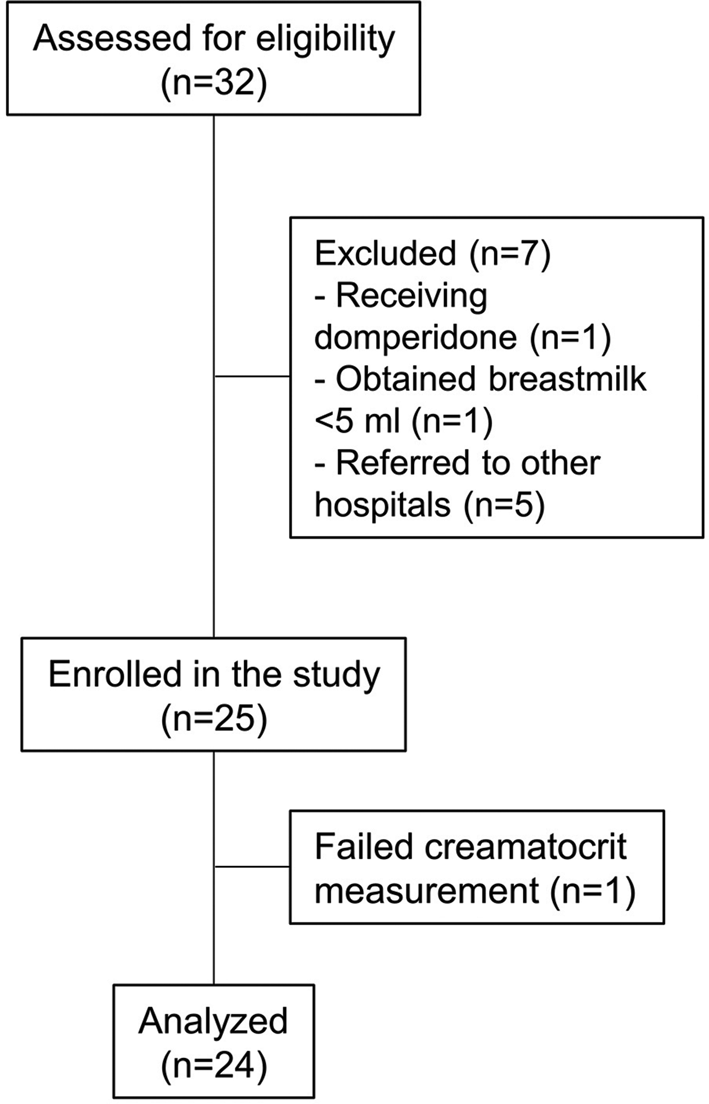
Flow diagram. Thirty-two participants were assessed for eligibility and 25 were enrolled. Sequential breastmilk samples were assessed for 24 participants, with one mother excluded from analyses due to unsuccessful creamatocrit measurements

Thirty-two mothers were assessed for eligibility, seven were excluded, and 25 were enrolled (Fig. 3). Mothers were recruited 15–70 days after childbirth (64% within 30 days). The mean (standard deviation [SD]) maternal age was 27.9 (6.3) years. Twelve out of 25 mothers had underlying diseases or complications during pregnancy; this included: twin pregnancies (n = 5), pregnancy-induced hypertension (PIH, n = 4), and history of thyrotoxicosis (n = 3). The four mothers with PIH were clinically stable and did not receive any medication during the study period. The three mothers with a history of thyrotoxicosis were in a euthyroid state and did not receive any medication as well. Fourteen mothers (58%) had breastfeeding experience.

Many mothers (87.5%) exhibited a disparity in expressed breastmilk between their left and right breasts (Table 2). No significant difference in breastmilk volume was observed between mothers with and without breastfeeding experience (mean [SD] of 63.5 [44.2] mL vs. 55.4 [23.6] mL, respectively [t = 0.59, df = 22,*p* = 0.56])

**Table 2:** Expressed breastmilk per breast and total breastmilk volume from each mother

	n	Median	IQR	Min, Max
Expressed breastmilk per breast (mL)	48	25	15, 35	5, 90
Total breastmilk volume from each mother (mL)	24	55	35, 75	20, 170
Difference between left and right breasts (mL)	24	5	5, 10	0, 35

Numbers represent data from 24 mothers (48 breasts). Abbreviations: IQR, interquartile range; Max, maximum; Min, minimum; n, number

The last aliquot of breastmilk had a significantly higher creamatocrit compared with the first aliquot from the same breast (median [IQR] of 12.7% [8.9%, 15.3%] and 5.6% [4.3%, 7.7%], respectively [test statistic 1128, Wilcoxon signed-rank test; *p* < 0.001])

**Fig. 4:**
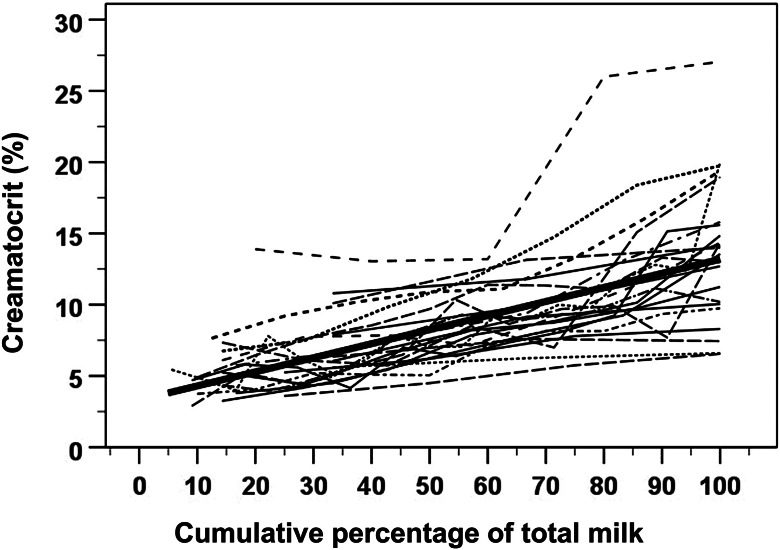
Relationship between creamatocrit (%) and the cumulative percentage of total milk volume (CP-MV). A spaghetti plot was used to visually explore the relationship between creamatocrit and CP-MV. The bold line represents the fit regression line from the final mixed model (Model 2.1)

Data from 23 mothers were included in the random-coefficient mixed model analysis, as one mother expressed less than three bottles of breastmilk per breast. Eleven mothers provided left-breast and 12 provided right-breast data. 155 aliquots of breastmilk were collected, with a range of 3–18 bottles per breast (mean [SD] of 6.7 [3.7] bottles). The results of fitting the four mixed models are summarized in Table 3. Model 2 – with linear, random intercept and slope – was selected as the final model as it had the smallest AIC and BIC. However, Model 2 centered CP-MV (Xc), which was impractical. Model 2.1 (Table 3) considers CP-MV (X) as an independent variable and was fitted to obtain the following final mixed model equation Y = 3.3157 + 0.09874 × X – where Y represented creamatocrit and X represented CP-MV. The creamatocrit increased by 1% for every 10% incremental increase in expressed breastmilk volume until the breast was emptied (Fig. 4)

**Table 3:** Random-coefficient mixed model analysis results

Model	-2 log likelihood	AIC	BIC	Regression equation Y = b_0_ + b_1_ × X + b_2_ × X^2^	*p*-value		
					b_0_	b_1_	b_2_
1	699.7	703.7	706.0	Y = 8.3047 + 0.09863 × Xc	< 0.001	< 0.001	-
2	670.4	678.4	682.9	Y = 8.2525 + 0.09874 × Xc	< 0.001	< 0.001	-
3	706.7	710.7	713.0	Y = 7.8581 + 0.09019 × Xc + 0.00058 × Xc^2^	< 0.001	< 0.001	0.004
4	673.6	681.6	686.2	Y = 7.8134 + 0.08553 × Xc + 0.00066 × Xc^2^	< 0.001	< 0.001	0.002
2.1	670.4	678.4	682.9	Y = 3.3157 + 0.09874 × X	< 0.001	< 0.001	-
				SE(b_1_) = 0.008011 95% CI of β_1_ = (0.08212, 0.1154)			

The mean cumulative percentage of total milk volume (CP-MV, represented by X) from 155 bottles was 57%. A value of 50% was used to center X (represented by Xc). The relationship between X and Xc can be expressed as Xc = X – 50. Creamatocrit was represented by Y. Abbreviations: AIC, Akaike information criteria; BIC, Bayesian information criteria; CI, confidence interval; SE, standard error; Xc, centered X

Upon comparing the creamatocrit of different foremilk:hindmilk ratios, the mean creamatocrit in hindmilk portions (9.23%, 9.35%, 9.81%, and 10.62%, respectively) was significantly higher than foremilk portions (6.28%, 6.33%, 6.72%, and 7.17%, respectively) at all predefined ratios (20:80, 25:75, 33:67, and 50:50) (Supplementary Table S1).

Post hoc analyses were performed by grouping the expressed breastmilk volume from each breast into high-volume (> 25-mL) and low-volume (≤ 25-mL) groups (using the median 25-mL observed in the study as a reference volume). Low-volume mothers had a significantly higher creamatocrit in the first aliquot of breastmilk compared with high-volume mothers (*p =* 0.002, Table 4). The opposite was true for high-volume mothers (*p* = 0.005, Table 4).

**Table 4:** Creamatocrit of the first and last breastmilk aliquots in high-volume and low-volume groups

Creamatocrit (%)	Group		U	*p*-value
	High-volume (n = 23)	Low-volume (n = 25)		
First aliquot, median (IQR)	4.7 (3.8, 5.9)	6.4 (5.4, 9.7)	437	0.002
Last aliquot, median (IQR)	14.4 (11.1, 15.9)	9.83 (7.4, 14.1)	144	0.005
Difference between aliquots, median (IQR)	9.3 (6, 11.6)	3.2 (1.4, 3.9)	53	< 0.001

High-volume refers to breastmilk volumes > 25-mL per breast. Low-volume refers to breastmilk volumes ≤ 25-mL. A Mann-Whitney U test was used to compare creamatocrit between the first and last aliquots of high-volume and low-volume groups due to the non-normal distribution of data (test values are indicated by U). Abbreviations: IQR, interquartile range; mL, milliliter

## Discussion

This study characterized the change in breastmilk’s fat content and explored the feasibility of defining a demarcation between foremilk and hindmilk from the start to end of milk flow. The researchers found that the fat content in breastmilk linearly increased towards the end of the milk stream (Fig. 4). It demonstrated a positive correlation between creamatocrit and subsequent aliquots of breastmilk collected over time (represented by CP-MV; Fig. 4). Regardless of the foremilk-to-hindmilk ratio, creamatocrit was consistently higher in portions of hindmilk compared with foremilk (1.5 times as high in the last than the first aliquots; Supplementary Table S1).

The positive correlation between CP-MV and creamatocrit observed in this study reaffirms previous observations that hindmilk contains a higher fat concentration than foremilk [11, 16, 19, 20, 26]. This progressive increase is explained by the increased number of milk fat globules released into breastmilk as the mammary lobe is emptied [27]. This study further explicated the correlation between creamatocrit and CP-MV to be a 1% increase in fat content for every 10% incremental increase in expressed breastmilk volume until the breast is emptied (Table 3, Model 2.1).

The observed incremental increase in breastmilk’s fat content also suggests that there is no exact point of demarcation between foremilk and hindmilk. For low-volume mothers, conventional definitions of foremilk could lead to breastmilk with high-fat content being mistakenly discarded – as a significantly higher creamatocrit was found in the first aliquot of breastmilk. For high-volume mothers, it is important to avoid overfeeding infants with foremilk, as a significantly higher creamatocrit is found in breastmilk expressed towards the end of the milk stream. This highlights the importance of understanding the typical breastmilk volume a mother expresses and that infants can consume to ensure a fat-rich portion can be sufficiently obtained for VLBW infants. The researchers suggest that a more fluid definition of foremilk and hindmilk be adopted. This would enable clinicians to capture as much of hindmilk’s nutritional and caloric values as possible for infants with limited feeding volumes – preventing poor growth outcomes. It is also crucial to emphasize the importance of fully emptying the breast during breastmilk expression. This both increases breastmilk volume by removing the feedback inhibitor of lactation as well as optimizes fat-rich hindmilk collection [28].

Mothers can prepare their breastmilk into aliquots based on the required feeding volume of their infant(s). However, caution should still be exercised when prioritizing hindmilk over foremilk. For example, retinol and alpha-tocopherol were found in higher concentrations in hindmilk than foremilk [16]. In addition, despite the significantly greater total protein content in hindmilk, foremilk contains significantly higher total free amino acids [19]. The physiological importance of the different nutritional components of foremilk and hindmilk requires further investigation. Once able, infants should consume breastmilk from the start to end of milk flow to ensure sufficient nutrition and calories required for growth.

Previous literature found that mothers with previous breastfeeding experience expressed a greater volume of breastmilk than mothers with no experience [17, 29]. This could be explained by the greater number of prolactin receptors present in the mammary glands of multiparous compared with primiparous mothers [29]. This study found no association between breastfeeding experience and expressed breastmilk volume. However, the breastmilk analyzed in this study was collected from a single pumping session, and therefore, may not be representative of the total breastmilk volume expressed each day [30]. In addition, several factors affect breastmilk production, for instance: childbirth type, onset of lactogenesis II, frequency of breastmilk expression, breast pump type, underlying disease(s), and mothers’ concomitant medication [31–33]. However, analyzing the influence of such factors was beyond the scope of this study. The disproportionate number of women with previous thyrotoxicosis is likely due to under-treated hypothyroidism or their underlying autoimmune conditions taking their natural course, resulting in premature childbirths.

This study had some limitations. First, the lack of data on maternal nutrition that may affect the nutritional compositions of breastmilk. Second, creamatocrit measurements were performed by a single researcher. While there is a risk of systematic measurement errors, this was highly unlikely due to the extensive training undergone by the researcher before measurements were performed. Third, the sample size was limited by resources and calculations were performed post hoc. Fourth, this was a single-center study that included mothers of very preterm infants. The findings of this study may not be generalizable to late preterm or full-term infants. Fifth, the disparity in infants’ age. However, this disparity was not a primary concern of this study, as it focused on the change in fat concentration from the start to end of milk flow. Hence, any variability in fat content found across a range of ages was welcomed. Additionally, mothers were recruited at least two weeks after childbirth in order to obtain mature milk, which already has less variation in fat concentration compared with the first two weeks after childbirth [34].

## Conclusions

The fat content in breastmilk increases from the start to end of milk flow. To ensure optimal nutrition for an infant, practitioners should recommend individualized feeding regimens and adopt more fluid definitions of foremilk and hindmilk. Mothers may also prepare their breastmilk in aliquots based on the required feeding volume of their infant, prioritizing aliquots of hindmilk before foremilk to ensure optimal caloric intake. Hindmilk feeding regimens warrant further nutritional considerations

### Electronic supplementary material

Below is the link to the electronic supplementary material.

Supplementary Material 1

## Data Availability

The data sets used and/or analyzed in the current study are available from the corresponding author upon reasonable request.

## Abbreviations

AIC: Akaike information criteria
BIC: Bayesian information criteria
CI: confidence interval
COA: certificate of approval
CP-MV: cumulative percentage of total milk volume
IQR: interquartile range
mL: milliliter
µL: microliter
NICU: neonatal intensive care unit
SD: standard deviation
VLBW: very low birth weight
wk: week
