# Supplementary material for: The relationship between creamatocrit and cumulative percentage of total milk volume: a cross-sectional study in mothers of very preterm infants in Bangkok, Thailand
Source: Int Breastfeed J. 2023 Nov 23;18:63. doi: 10.1186/s13006-023-00599-5 (PMC10668363; doi:10.1186/s13006-023-00599-5)
Supplement: Supplementary file 1 — Supplementary Material 1 [file 13006_2023_599_MOESM1_ESM.docx]

**Supplementary Results**

**The Relationship Between Creamatocrit and Cumulative Percentage of Total Milk Volume: A Cross-Sectional Study in Mothers of Very Preterm Infants in Bangkok, Thailand**

| **Table of Contents** | |
| --- | --- |
| 1. Supplementary Table 1. Creamatocrit difference between foremilk and hindmilk from the same breast. |  |

**Supplementary Table S1** Creamatocrit difference between foremilk and hindmilk from the same breast

| Foremilk: Hindmilk | Breasts (n) | Creamatocrit (%),  Mean ± SD | | | t | df | *p*-value |
| --- | --- | --- | --- | --- | --- | --- | --- |
|  |  | Foremilk | Hindmilk | Difference |  |  |  |
| 20:80 | 42 | 6.28±2.24 | 9.23±3.02 | 2.95±1.74 | 11.02 | 41 | <0.001 |
| 25:75 | 42 | 6.33±2.26 | 9.35±2.97 | 3.02±1.64 | 11.94 | 41 | <0.001 |
| 33:67 | 47 | 6.72±2.78 | 9.81±3.27 | 3.09±1.77 | 11.94 | 46 | <0.001 |
| 50:50 | 47 | 7.17±2.82 | 10.62±3.42 | 3.45±1.91 | 12.40 | 46 | <0.001 |

A cumulative percentage of total milk volume of 20%, 25%, 33%, and 50% were used to define the portion of foremilk. 20:80 and 25:75 foremilk:hindmilk ratios required at least three bottles of breastmilk for analyses. Six breasts expressed less than three aliquots of breastmilk and were therefore excluded. 33:67 and 50:50 foremilk:hindmilk ratios required at least two bottles of breastmilk for analyses. Only one breast expressed less than two bottles of breastmilk and was therefore excluded. *p*-values were determined using a paired t-test. Abbreviations: df, degrees of freedom; SD, standard deviation; t, test value of the paired t-test.
